# Asparaginase pharmacokinetics and implications of therapeutic drug monitoring

**DOI:** 10.3109/10428194.2014.1003056

**Published:** 2015-03-11

**Authors:** Barbara Asselin, Carmelo Rizzari

**Affiliations:** ^a^ Department of Pediatrics, Golisano Children's Hospital, University of Rochester School of Medicine, Rochester, NY, USA; ^b^ Department of Pediatrics, Pediatric Hematology-Oncology Unit, University of Milano-Bicocca, MBBM Foundation, San Gerardo Hospital, Monza, Italy

**Keywords:** Asparaginase, acute lymphoblastic leukemia, pharmacokinetics, hypersensitivity, silent inactivation, therapeutic drug monitoring

## Abstract

Asparaginase is widely used in chemotherapeutic regimens for the treatment of acute lymphoblastic leukemia (ALL) and has led to a substantial improvement in cure rates, especially in children. Optimal therapeutic effects depend on a complete and sustained depletion of serum asparagine. However, pronounced interpatient variability, differences in pharmacokinetic properties between asparaginases and the formation of asparaginase antibodies make it difficult to predict the degree of asparagine depletion that will result from a given dose of asparaginase. The pharmacological principles underlying asparaginase therapy in the treatment of ALL are summarized in this article. A better understanding of the many factors that influence asparaginase activity and subsequent asparagine depletion may allow physicians to tailor treatment to the individual, maximizing therapeutic effect and minimizing treatment-related toxicity. Therapeutic drug monitoring provides a means of assessing a patient's current depletion status and can be used to better evaluate the potential benefit of treatment adjustments.

## Introduction

Over the past 50 years, asparaginase (ASP) has become a key component of treatment protocols for acute lymphoblastic leukemia (ALL). Lymphoblastic leukemic cells lack or have low levels of the enzyme asparagine synthetase; therefore, they are unable to produce asparagine on their own and heavily depend on extracellular sources [1]. At sufficient activity levels, ASP depletes serum asparagine and eventually leads to leukemic cell death [1]. With the addition of ASP to existing chemotherapeutic regimens, long-term survival rates in children with ALL have improved; however, significant challenges still exist in optimizing treatment for many patients [2,3].

While asparagine depletion is considered the pharmacological goal of ASP therapy, there is no universally accepted dose or treatment schedule for all patients. Asparagine levels vary greatly between individuals, with several factors influencing the relationship between ASP dose and serum asparagine concentrations. Optimal treatment necessitates practitioners to be knowledgeable of the basic pharmacological principles underlying ASP therapy and utilize this knowledge to effectively guide patient care. The goal of this review is to give a practical overview of the pharmacology underlying ASP therapy in the treatment of patients with ALL.

## The role of asparagine and glutamine depletion

**Figure 1.**
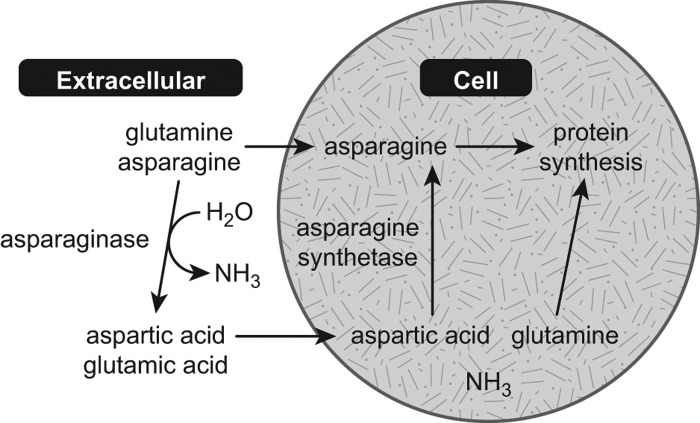
Mechanism of action of asparaginase [1]. Adapted with permission from Muller and Boos, 1998 [1].

Asparagine is a non-essential amino acid and can be synthesized from aspartic acid in healthy cells, or obtained from the diet. Cellular synthesis of asparagine is accomplished via the enzymatic action of asparagine synthetase (Figure 1). Insufficient levels of cellular asparagine lead to reduced DNA, RNA and protein synthesis; inhibition of cell growth; and ultimately the activation of apoptotic cell-death mechanisms [1].

Lymphoblastic leukemic cells do not express asparagine synthetase; therefore, they are unable to synthesize asparagine *de novo*. Instead, leukemic cells rely on exogenous asparagine to fuel protein synthesis and cell growth. ASP achieves its effect by depleting circulating asparagine concentrations, selectively targeting leukemic cells. The complete and sustained depletion of asparagine is believed to be critical to the long-term success of ASP therapy. Under normal physiological conditions, circulating asparagine concentrations range between 40 and 80 μM [4,5]. Although no formal criterion exists, researchers have previously defined complete asparagine depletion as less than 0.1–0.2 μM based on the limit of detection of the high-performance liquid chromatography assay used [4–6]. However, the critical level of asparagine depletion in serum required for *in vivo* leukemic cell death is unknown.

Results from several studies highlight the importance of asparagine depletion in both pediatric and adult patients with ALL [7–9]. Jarrar *et al*. showed an inverse relationship between day 14 asparagine concentrations and remission rate on day 35 in 214 children with first relapse of ALL [7]. Patients with serum asparagine levels < 1 μM were more likely to achieve second remission compared with patients with higher asparagine levels.

Currently available ASP formulations exhibit preferential selectivity for asparagine, but also hydrolyze glutamine to a lesser extent (Figure 1) [10]. Given that glutamine can be used as an amino-group donor in the synthesis of asparagine [11], it has been suggested that depletion of both asparagine and glutamine are necessary to achieve optimal outcomes [12,13]. Recent experimental evidence in acute myeloid leukemia cell lines suggests that the therapeutic effect of ASP may largely be due to its effects on intracellular glutamine and inhibition of downstream mammalian target of rapamycin complex 1 (mTORC1) signaling pathways [14]. It has been reported that > 90% deamination of glutamine levels is needed to achieve optimal asparagine depletion *in vivo* [13]. Current formulations of ASP differ in their glutamine pharmacokinetics. While both *Erwinia chrysanthemi*- and *Escherichia coli*-derived ASP formulations show similar binding affinities (K_m_) for glutamine, the maximal conversion rate at saturation (K_cat_) is greater with ASP *E. chrysanthemi* (Table I) [10,15–17]. For all formulations, however, selectivity for glutamine is markedly weaker than is found with asparagine. Furthermore, glutamine levels in blood are much higher than asparagine levels, and a relatively greater ASP activity level is needed to sufficiently reduce levels of both amino acids [12].

**Table I.** Biochemical properties of asparaginase with regard to asparagine and glutamine [10]*.

Source organism	Asparagine		Glutamine		References
	K_m_ (mM)	K_cat_ (s^− 1^)	K_m_ (mM)	K_cat_ (s^− 1^)	
*Erwinia chrysanthemi*	0.058–0.080	397–440	1.7–6.7	65–72	[15,16]
*Escherichia coli*	0.015	24	3.5	0.33	[17]

K_m_, binding affinity; K_cat_, maximal conversion rate at saturation.

*Adapted with permission from Covini *et al*., 2012 [10].

## The use of asparaginase in acute lymphoblastic leukemia

Currently, three different types of ASP are available for use in the treatment of ALL. Native *E. coli* ASP and pegylated (PEG)-ASP are derived from the bacteria *E coli*. ASP *E. chrysanthemi* is derived from *E. chrysanthemi.* It has a distinct immunogenic profile, making ASP *E. chrysanthemi* an appropriate treatment option for patients who experience hypersensitivity to *E. coli*-derived formulations [18,19]. Native *E. coli* ASP is no longer available in the United States [20], and is being replaced by PEG-ASP and ASP *E. chrysanthemi* in new protocols.

Activity levels of ASP inversely correlate with serum asparagine concentrations, and are commonly used as a proxy measure to estimate asparagine depletion [4,9,11,21–23]. Early experiments in non-human primates indicate that asparagine depletion in the serum and central nervous system consistently occurs at ASP activity ≥ 0.1 IU/mL [21]. This 0.1 IU/mL target has subsequently received support from a number of human trials [4,24,25], and is generally accepted as the activity level necessary to achieve therapeutic depletion of asparagine [2].

Several studies show an association between ASP activity and positive outcomes in patients with ALL [26–29]. A study conducted in adults by the Cancer and Leukemia Group B compared outcomes between patients treated with PEG-ASP with ASP activity > 0.03 IU/mL and patients with activity < 0.03 IU/mL [26]. Overall, the 63 patients with ASP activity > 0.03 IU/mL showed greater median survival compared with the 22 patients with reduced activity, 31 vs. 13 months, respectively (*p* = 0.001). A prolonged course of high-dose intensity, likely resulting in prolonged asparagine depletion, has also been shown to improve outcomes in children with ALL [27–29].

## Relationship between dose, asparaginase activity and depletion of asparagine

A number of factors influence ASP activity and asparagine concentrations following a given ASP dose. The formulation of ASP, degree of interpatient variability, formation of ASP antibodies, concomitant medications and even the method of administration can have an important impact on ASP activity dynamics and patient outcomes.

### Asparaginase formulations

All three ASP formulations show equivalent leukemic cell kill *in vitro* [23]. However, the pharmacokinetic properties of each ASP differ greatly (Table II) [30,31]. PEG-ASP shows the longest half-life of the three formulations, which has been estimated at 5.7 days following intramuscular (IM) administration. ASP *E. chrysanthemi* shows the shortest half-life at approximately 15.6 h [31]. These differences carry practical implications for constructing optimal dose schedules, as formulations with longer half-lives are cleared at a slower rate, and therefore provide relatively longer exposure to the enzyme and subsequent duration of asparagine depletion. For this reason, different ASP preparations are not readily interchangeable. Identifying the appropriate dose schedule to achieve therapeutic levels of ASP activity for the different ASP formulations has been the focus of numerous studies (Table III) [4,5,8,9,22,32–36].

**Table II.** Pharmacokinetic characteristics of the three asparaginase formulations [30,31].*

	*Erwinia asparaginase*	Native *E. coli* asparaginase	PEG-asparaginase
Half-life (mean ± SD)	0.65 ± 0.13 days	1.28 ± 0.35 days	5.73 ± 3.24 days
Asparagine depletion	7–15 days	14–23 days	26–34 days
Peak asparaginase activity	Within 24 h	24–48 h	72–96 h

SD, standard deviation; PEG, pegylated.

*Adapted with permission from Asselin, 1999 [30].

**Table III.** Selected pharmacokinetic studies [4,5,8,9,22,32–36].

Type of asparaginase	Patients	Key pharmacokinetic results	Reference
PEG-ASP, ASP *Erwinia chrysanthemi*	89	• PEG-ASP IV (2500 IU/m^2^ every 2 weeks) resulted in mean ASP activity of 0.9 IU/mL • All patients with clinical hypersensitivity (22%) showed activity levels of 0 IU/mL	Tong 2014 [32]
ASP *Erwinia chrysanthemi*	58	• ASP *Erwinia chrysanthemi* 25 000 IU/m^2^ IM administered on a M/W/F schedule achieved therapeutic NSAA* for the majority of patients at 48 and 72 h post-dose	Salzer 2013 [33]
ASP *Erwinia chrysanthemi*	38	• Median NSAA* was 0.247 IU/mL 3 days and 0.077 IU/mL 4 days after ASP *Erwinia chrysanthemi* 25 000 IU/m^2^ IM twice weekly	Vrooman 2010 [34]
Native *E. coli* ASP, PEG-ASP	118	• PEG-ASP 2500 IU/m^2^ IM provided activity > 0.03 IU/mL for 15–21 days • ASP activity > 0.03 IU/mL for a greater number of days in patients administered PEG-ASP compared with patients treated with 6000 IU/m^2^ IM native *E. coli* ASP on M/W/F	Dinndorf 2007 [8]
PEG-ASP	55	• Single dose of PEG-ASP 2000 IU/m^2^ in newly diagnosed adults with ALL resulted in complete asparagine deamination in 100% of patients at 2 h and 81% at 21 days • Elimination half-life of 7 days	Douer 2007 [9]
PEG-ASP	20	• 1000 IU/m^2^ IV every 2 weeks during induction and once during reinduction resulted in adequate serum ASP activity and asparagine depletion	Rizzari 2006 [35]
Native *E. coli* ASP, PEG-ASP	118	• Patients randomized to 2500 IU/m^2^ PEG-ASP or 6000 IU/m^2^ native *E. coli* ASP • Half-life of PEG-ASP was 5.5 days and 26 h for native *E. coli* ASP • Serum ASP activity and levels of asparagine or glutamine inversely correlated	Avramis 2002 [22]
ASP *Erwinia chrysanthemi*	40	• 30 000 IU/m^2^ IM every day resulted in 92% of patients with NSAA* ≥ 0.5 IU/mL • 30 000 IU/m^2^ IM twice a week resulted in 73% of patients with NSAA* ≥ 0.1 IU/mL	Albertsen 2001 [36]
ASP *Erwinia chrysanthemi*	21	• ASP *Erwinia chrysanthemi* 20 000 IU/m^2^ IV on a M/W/F schedule • Mean 48 h trough ASP activity level was 0.16 ± 0.099 IU/mL (mean ± SD) • Mean 72 h trough ASP activity level was 0.05 ± 0.039 IU/mL (mean ± SD)	Vieira Pinheiro 1999 [5]
Native *E. coli* ASP (medac^®^), native *E. coli* ASP (Crasnitin^®^), ASP *Erwinia chrysanthemi*	56	• Single dose of 10 000 IU/m^2^ ASP medac resulted in greater 3-day post-dose activity compared with Crasnitin and ASP *Erwinia chrysanthemi* • Higher median ASP levels are more likely to result in asparagine depletion	Boos 1996 [4]

ASP, asparaginase; IM, intramuscular; IV, intravenous; M/W/F, Monday/Wednesday/Friday; PEG, pegylated (polyethylene glycol); NSAA, nadir serum asparaginase activity; SD, standard deviation.

*Therapeutic NSAA is ≥ 0.1 IU/mL.

Identical dose schedules of *E. coli-*derived formulations and ASP *E. chrysanthemi* can result in significantly different ASP activity levels and may lead to a worse outcome, thus giving the misleading perception of reduced efficacy [37,38]. Duval *et al.* compared outcomes in 700 children treated on identical dose schedules of ASP *E. chrysanthemi* or native *E. coli* ASP (10 000 IU/m^2^ administered twice weekly) [38]. ASP *E. chrysanthemi* was associated with significantly inferior 6-year event-free survival (EFS) and inferior overall survival. A study by Boos *et al*. compared trough asparagine and ASP activity levels in 49 children given the same 10 000 IU/m^2^ dose of three ASP preparations (ASP medac^®^, Crasnitin^®^ and ASP *E. chrysanthemi*) [4]. The investigators found lower mean ASP activity for patients receiving ASP *E. chrysanthemi* compared with those treated with the same dose of native *E. coli* ASP (ASP medac or Crasnitin) [4]. Correspondingly, the percent of subjects achieving complete asparagine depletion (defined as ≤ 0.1 μM) was also lower in patients receiving ASP *E. chrysanthemi* [4]. Additional evidence suggests that ASP *E. chrysanthemi* may be associated with favorable results if administered at a higher dose and greater frequency in specific treatment paradigms [33,34]. In the Associazione Italiana Ematologia Oncologia Pediatrica (AIEOP) ALL 95 study protocol, standard-risk (SR) patients received reduced-intensity treatment compared with patients in the intermediate-risk (IR) group. Both risk groups were randomized to receive either standard treatment or extended intensification with ASP *E. chrysanthemi* (25 000 IU/m^2^ given IM weekly for 20 weeks) during maintenance in SR and during reinduction and maintenance in IR patients. Patients treated with prolonged high-dose ASP therapy showed significantly improved outcomes compared with those treated according to the standard ASP regimen in the SR group (10-year disease-free survival of 78.7% and 87.5%, respectively) but not in the IR group [28,39]. Taken together, these results suggest that earlier reports of suboptimal outcomes with ASP *E. chrysanthemi* are most probably the result of inadequate dosing and the subsequent lower levels of ASP activity in those patients.

Due to its shorter half-life, ASP *E. chrysanthemi* must be administered at higher doses and/or more frequently than PEG-ASP or native *E. coli* ASP in order to maintain consistent asparagine depletion. Albertsen *et al.* found that when ASP *E. chrysanthemi* was administered at 30 000 IU/m^2^ intravenously (IV) or IM twice weekly, target ASP activity levels of ≥ 0.1 IU/mL were achieved in approximately two-thirds of samples [36]. Results of the Children's Oncology Group (COG) ALL07P2 trial showed that 25 000 IU/m^2^ ASP *E. chrysanthemi* administered IM three times a week achieved ASP activity > 0.1 IU/mL in 92.7% of evaluable patients at 48 h and 88.4% of evaluable patients at 72 h post-dose [33]. Based on these results, the substitution dose of ASP *E. chrysanthemi* recommended by the Food and Drug Administration (FDA) in patients with hypersensitivity to *E. coli*-derived ASP is 25 000 IU/m^2^ administered IM for each scheduled dose of native *E. coli* ASP three times a week (Monday/Wednesday/Friday) for six doses for each planned dose of PEG-ASP [33,40]. The current AIEOP–Berlin, Frankfurt, Münster (BFM) ALL 2009 trial includes a randomized intensification study of extended-use PEG-ASP. A dose of 2500 IU/m^2^ has been carefully chosen to adequately replace the dose intensity provided by previously used native *E. coli* products [41]. Furthermore, the strict implementation of therapeutic drug monitoring (TDM) to identify patients with suboptimal ASP activity has been incorporated into this study since 2009. Patients with no observable ASP activity or overt signs of clinical hypersensitivity are switched to ASP *E. chrysanthemi*. In the AIEOP– BFM ALL 2009 protocol, the substitution dose for a single administration of PEG-ASP is seven doses of 20 000 IU/m^2^ ASP *E. chrysanthemi* administered IV every other day.

### Interpatient variability

A number of studies report pronounced variability in ASP activity measurements between patients on identical treatment schedules [5,42,43]. One study found widespread interpatient variability in the ASP activity of 763 patients administered three different ASP preparations. The largest group of patients (*n* = 416) were administered 1000 IU/m^2^ PEG-ASP IV [42]. While median ASP activity for these patients was well above the 0.1 IU/mL criterion, the researchers observed pronounced interpatient variability, with ASP activity levels between 0 IU/mL and 3.3 IU/mL within 7 days of dose administration. Overall, the researchers found 30% of ASP activity samples < 0.1 IU/mL. A second study focused on ASP activity in patients switched to 20 000 IU/m^2^ ASP *E. chrysanthemi* IV administered on a Monday/Wednesday/Friday schedule [5]. The researchers reported substantial interpatient variability in ASP activity at days 2 and 3 post-treatment. Trough ASP activity ranged between < 0.02 IU/mL and > 0.5 IU/mL in the 21 patients for which data were available. Recently reported results of 25 000 IU/m^2^ ASP *E. chrysanthemi* administered IV in patients with hypersensitivity to *E. coli*-derived ASP highlight pronounced variability in ASP activity between patients [43]. In this study, the coefficient of variation of ASP activity measured 48 and 72 h post-dose ranged from 72% to 109%.

### Asparaginase antibody formation

ASP is a non-human protein, and has the potential to elicit an immune response in patients. The formation of ASP antibodies can substantially reduce ASP activity and negatively impact outcomes [18,44]. The presence of ASP antibodies is often associated with symptoms of clinical hypersensitivity [18,45]. However, a number of patients develop antibodies without any outward signs of allergy, referred to as silent inactivation or subclinical hypersensitivity [5,30]. Subclinical hypersensitivity was found to occur in 29% of patients with ALL during front-line treatment with native *E. coli* ASP in one large monitoring study [18]. The risk of subclinical hypersensitivity at the time of relapse is even higher; therefore, it should be considered in designing treatment regimens for relapsed disease [46,47]. This condition presents a serious challenge to achieving ASP depletion in many patients, with a number of reports recommending the regular monitoring of ASP activity as a means of identifying patients with subclinical hypersensitivity [5,42,48].

Different ASP formulations each have a distinct immunogenic profile and are associated with a different risk of antibody formation. The incidence of antibodies in patients treated with native *E. coli* ASP has been estimated as high as 60% during the entire course of therapy [18]. Antibody formation or hypersensitivity with either ASP *E. chrysanthemi* or PEG-ASP is less prevalent, with studies reporting antibodies in 2–18% of patients treated with PEG-ASP [22,49] and 8–33% with ASP *E. chrysanthemi* [50,51]. PEG-ASP is derived from the same bacterial strain as native *E. coli* ASP, and patients often exhibit significant cross-resistance between these formulations [18,47,52,53]. In a recent study, researchers evaluated the cross-reactivity of samples drawn from 16 patients who developed native *E. coli* ASP antibodies during reinduction [53]. None of these samples showed any reactivity with ASP *E. chrysanthemi*; however, 63% of samples (10 of 16) showed a significant reaction to PEG-ASP.

A number of strategies have been suggested to reduce the risk of antibody formation. These strategies include: (1) pretreatment with glucocorticoids; (2) treating patients on an intense and continuous dose schedule; and (3) switching to an alternative ASP when appropriate.

A comparison across studies shows that clinical hypersensitivity is most common when ASP is given without concurrent steroids, suggesting that the immunosuppressive effect of steroids might decrease the immunogenic risk [18,22,51,54]. However, the use of corticosteroids may also mask the presence of subclinical hypersensitivity, which is associated with significantly reduced ASP activity levels.

The consistency and intensity of ASP therapy may also influence the risk of antibody formation. Studies show a reduced incidence of ASP antibodies in protocols where ASP was administered at a greater frequency and with fewer gaps between treatment periods [36,52]. It has been suggested that large concentrations of ASP may overwhelm the immune response and lower the likelihood of future hypersensitivity [12,36].

When clinical hypersensitivity or subclinical hypersensitivity is observed, patients should be immediately switched to a different ASP formulation. Improved outcomes are seen when subclinical hypersensitivity can be rapidly identified and appropriate treatment adjustments are made [48].

A recent study by Tong *et al*. focused on improving therapeutic benefits from ASP therapy by reducing the risk of hypersensitivity or subclinical hypersensitivity in children with ALL [32]. Patients were treated with PEG-ASP during consolidation, after receiving native *E. coli* ASP during induction. The authors reported high PEG-ASP activity levels in patients without clinical hypersensitivity (70% of the study population); however, 22% of patients developed allergy, and 8% showed subclinical hypersensitivity [32]. Importantly, patients with hypersensitivity or subclinical hypersensitivity showed no asparagine depletion and substantially lower ASP activity levels compared with patients who did not show an immune response [32]. These findings partially confirm the results of an earlier investigation using a different treatment schedule, and highlight the utility of trough ASP activity measurement to identify patients with subclinical hypersensitivity [55].

### Route of administration

In Europe, ASP is commonly administered IV, while IM is the approved method of administration in the United States. Early studies indicated that IM administration was associated with a lower incidence of immune reaction [56]; however, a number of recent reports show comparable rates with both methods of administration [9,12,22,27,45]. In fact, IV administration may be preferable in some instances, such as in the case of a severe anaphylaxis, as infusion of the drug can be stopped immediately, sparing the patient from full exposure, whereas this is not possible with IM injections. Administration by IV also allows the use of high-continuous-dose schedules without the pain and anxiety of numerous IM injections.

Following IV administration, the observed time to peak activity of ASP is much shorter, and may result in decreased time of exposure (area under the curve) compared with IM injections [57]. The elimination half-life of IV-administered ASP *E. chrysanthemi* was reported as 6.4 h in one study [57], while the half-life following IM injection of ASP *E. chrysanthemi* has been shown to be over 15 h [31]. Similar differences have been found with *E. coli*-derived formulations, with estimates ranging from 24 to 32 h [31,58]. The longer half-life reported with IM administration is largely a factor of the slow absorption rate following injections, limiting the rate of enzyme elimination. Due to this, the decline in plasma activity following IM injections represents both the absorption and elimination of the enzyme [1,57]. Furthermore, IV administration of ASP is associated with significantly higher peak enzymatic activity levels and lower trough activity compared with an identical IM dose [42,57]. These differences can have practical implications for asparagine depletion. A recent study evaluated trough ASP activity levels following an identical dose of ASP *E. chrysanthemi* 10 000 IU/m^2^ administered either IV or IM in patients with ALL during reinduction [42]. The researchers found a greater percentage of patients with trough activity ≥ 0.1 IU/mL in the IM group compared with the IV group (IM, 85%; IV, 55%). These results highlight the need to account for pharmacokinetic differences between routes of drug administration when constructing treatment schedules. When ASP is administered IV, the frequency or dose may need to be increased to ensure continuous asparagine depletion.

## Therapeutic drug monitoring and clinical implications

TDM in ALL refers to the regular measurement of asparagine concentrations, ASP activity or ASP antibodies as a means of evaluating a patient's current treatment schedule. Subclinical hypersensitivity and interpatient variability can result in suboptimal ASP activity and negatively impact outcomes, yet are not directly addressed in many treatment protocols [34,48]. TDM provides a means of identifying patients with suboptimal ASP activity and offers physicians the necessary information to make informed adjustments in treatment.

A number of recent publications highlight the use of TDM in patients with ALL [5,36,42,48]. Of particular interest, Vrooman *et al*. report the results of a randomized clinical trial using TDM to guide individualized dose adjustment in patients undergoing ASP therapy [48]. In this study, patients were randomized into either a fixed-dose (FD) or an individualized-dose (ID) group. Patients in the FD group were administered 25 000 IU/m^2^ native *E. coli* ASP, while patients in the ID group began therapy on a lower dose, but were evaluated every 3 weeks for the need to adjust treatment. The goal of ID was to maintain trough ASP activity between 0.1 IU/mL and 0.14 IU/mL. The study found significantly higher 5-year EFS in the ID group (90%) compared with the FD group (82%, *p* = 0.04), likely due to the ability to identify and adjust treatment for ID patients who developed subclinical hypersensitivity, as patients with clinical hypersensitivity switched preparations in both groups. A total of 19 patients (10%) in the ID group showed subclinical hypersensitivity and were subsequently switched to a different ASP formulation (EFS, 95%). Following this change, 89% showed at least one trough ASP activity measurement ≥ 0.1 IU/mL. In the FD group, 18 patients (9%) showed no measurable ASP activity ≥ 0.1 IU/mL, with no overt signs of clinical hypersensitivity, and therefore were not switched to an alternative ASP (EFS, 76%).

Several European treatment protocols recommend the monitoring of ASP activity; however, TDM is not yet commonplace in the United States [2]. The FDA approval of ASP *E. chrysanthemi* as a component of multiagent chemotherapeutic regimens in patients with ALL who have developed hypersensitivity to *E. coli*-derived ASP was largely based on results from TDM studies [33,59]. In Europe, the Dutch Children's Oncology Group, the AIEOP and the BFM group recommend the periodic measurement of ASP activity and antibody levels in real time to modulate ASP treatment [32,41]. In these protocols, TDM is primarily used to determine subclinical hypersensitivity and evaluate the need to switch ASP formulations; however, the use of TDM as a dose-adjustment tool is increasing [32,41]. In the United States, a commercially available, Clinical Laboratory Improvement Amendments (CLIA)-certified, ASP activity assay has recently become available for use in patients undergoing ASP therapy with all currently approved ASP formulations [60].

## Conclusion

The complete and sustained depletion of asparagine is critical to achieving optimal outcomes with ASP therapy in the treatment of patients with ALL. Interpatient variability, the development of ASP antibodies, pharmacokinetic differences in ASP preparations and a host of other factors can affect the enzymatic activity following a given dose of ASP. To ensure optimal treatment, physicians must remain vigilant of these sources of variability and be ready to make any necessary treatment adjustments. As success rates in the treatment of ALL continue to increase, the goal can begin to shift toward improving the short-term and long-term quality of life of patients with ALL. Minimizing the number of long-term side effects, possibly through individually tailored treatment regimens, will be a key component toward achieving that goal.

Ongoing clinical studies promise to further clarify the relationship between ASP activity levels, outcomes and toxicity [41,61]. The increasing use of TDM will help identify patients with suboptimal enzyme activity and potentially inadequate asparagine depletion status. While there is a general consensus supporting the use of TDM to identify subclinical hypersensitivity, additional studies are needed to evaluate the benefits of TDM to guide dose adjustments in patients with measurable ASP activity. Additionally, a number of recent studies have reported promising results with ASP in the treatment of adolescent and young adult patients with ALL, a group with historically poor outcomes [62–66]. Future studies are needed to evaluate ASP pharmacokinetics and optimal treatment schedules in these older patients. These efforts will further contribute to shed light on what has recently been termed the “asparaginase galaxy” [67].
